# Review: Novel Insights Into Tumor Necrosis Factor Receptor, Death Receptor 3, and Progranulin Pathways in Arthritis and Bone Remodeling

**DOI:** 10.1002/art.39816

**Published:** 2016-11-28

**Authors:** Anwen Williams, Eddie C. Y. Wang, Lorenz Thurner, Chuan‐ju Liu

**Affiliations:** ^1^ Cardiff University Cardiff UK; ^2^ Saarland University Medical School Homburg, Saar Germany; ^3^ New York University Medical Center New York New York

## Introduction

Approximately 30 members of the tumor necrosis factor receptor superfamily (TNFRSF) have been identified. They are transmembrane proteins with cysteine‐rich motifs in their extracellular domains that bind to their cognate ligands 1. They are categorized into 3 groups: death domain–containing receptors, decoy receptors, and TNFR‐associated factor–binding receptors. Only 8 TNFRSF members contain a death domain (TNFR type I [TNFRI], death receptor 3 [DR‐3], DR‐4, DR‐5, DR‐6, Fas, nerve growth factor receptor, and ectodysplasin A receptor [EDAR]), of which TNFRI and DR‐3 constitute the principal focus of this article. Interactions between TNF superfamily (TNFSF) ligands and TNFRSF receptors help maintain tissue homeostasis by controlling survival, proliferation, differentiation, and effector function of immune cells. We limit our review to recent advances and novel insights into the roles of TNFRI and DR‐3 in bone and joint biology.

Bone cells (osteoblasts, osteoclasts, and osteocytes), fibroblast‐like synoviocytes, chondrocytes, and immune cells that infiltrate the arthritic joint will at different times express a wide range of TNFRSF members and TNFSF ligands. An overview of the current status of our knowledge in this regard is provided in Table 1. The impact of TNFRI activation on bone and inflammatory joint diseases has been researched in great depth 2, 3, but little or no data in the field have been reported on other more recently discovered TNFRSF members such as TROY (TNFRSF expressed on the mouse embryo; TNFRSF19), EDAR, and XEDAR (X‐linked ectodysplasin receptor; TNFRSF27). The unexpected interaction between progranulin (PGRN) and both TNFRI and TNFRII is particularly interesting in the context of arthritis‐associated bone pathology. PGRN levels are elevated in the synovial fluid of patients with rheumatoid arthritis (RA), osteoarthritis (OA), and other arthropathies 4, 5, 6, and PGRN has been shown to inhibit TNF‐induced osteoclastogenesis and promote osteoblast differentiation in mice 7. However, PGRN has a higher binding affinity for TNFRII (antiinflammatory with osteoprotective function) than for TNFRI (predominantly proinflammatory with degenerative function), which suggests conflicting actions. The potential overall impact of these divergent PGRN signaling pathways on the architecture of the arthritic joint has been evaluated 8.

**Table 1:** Cellular expression of death domain–containing TNFRSF members and their association with arthritis*

			Cells involved						
Receptor	Ligand	Association with arthritis	Osteoblasts	Osteoclasts	Osteocytes	Fibroblast‐like synoviocytes	Chondrocytes	Leukocyte subsets	References†
TNFRI (TNFRSF1A)	TNF (TNFSF2), LTα (TNFSF1), PGRN	RA, OA, SpA, arthropathies	Yes	Yes	Yes	Yes	Yes	All	2, 3, 4, 5, 6, 14, 77, 78
Fas (TNFRSF6)	FasL (TNFSF6)	RA, OA, arthropathies	Yes	Yes	Yes	Yes	Yes	All	79, 80, 81, 82
NGFR (TNFRSF16)	NGF	RA, OA, SpA, arthropathies	Yes	Yes	No	No	No	T cells	2, 79, 83
EDAR (TNFRSF27)	EDA	RA, arthropathies	No	No	No	No	No	Macrophage subsets	84
DR‐3 (TNFRSF25)	TL1A (TNFSF15), PGRN	RA, OA, SpA, arthropathies	Yes	Yes	No	No	No	CD4+ T cells, Treg cells, CD8+ T cells, IgM+ B cells, macrophages (inducible), neutrophils	10, 11, 12, 37, 50, 78
DR‐4 (TNFRSF10A)	TRAIL (TNFSF10)	RA, OA, SpA, arthropathies	Yes	Yes	No	Yes	Yes	Activated T cells	78, 79, 85, 86
DR‐5 (TNFRSF10B)	TRAIL (TNFSF10)	RA, OA, SpA, arthropathies	Yes	Yes	No	Yes	Yes	All	78, 85, 86
DR‐6 (TNFRSF21)	APP	None	Yes	Yes	No	No	Yes	T cells, B cells,dendritic cells	87

a TNFRSF = tumor necrosis factor receptor superfamily; LTα = lymphotoxin α; PGRN = progranulin; RA = rheumatoid arthritis; OA = osteoarthritis; SpA = spondyloarthritis; NGFR = nerve growth factor receptor; EDAR = ectodysplasin A receptor; DR‐3 = death receptor 3; TL1A = TNF‐like molecule 1A; APP = amyloid precursor protein.

The reference list presented in this table was limited by the requirements of the journal; as such, the citations for the expression of TNFRSF and TNFSF ligands by cells are not comprehensive.

DR‐3 and its TNFSF ligand TNF‐like molecule 1A (TL1A) contribute to the pathogenesis of autoimmune and rheumatic diseases 9; however, research in this area is very much in its infancy. Inhibition of DR‐3 reduces osteoclastogenesis and protects bones against the development of erosive pathology in experimental models of arthritis 10. A soluble form of DR‐3, produced by osteoblasts, regulates osteoblast apoptosis under tightly controlled conditions 11, 12. TL1A levels are elevated in serum from patients with RA compared with that from healthy controls. This review provides further insight into the role of DR‐3 in bone remodeling and arthritis.

## PGRN–TNFR interactions in arthritis and bone remodeling

**Figure 1:**
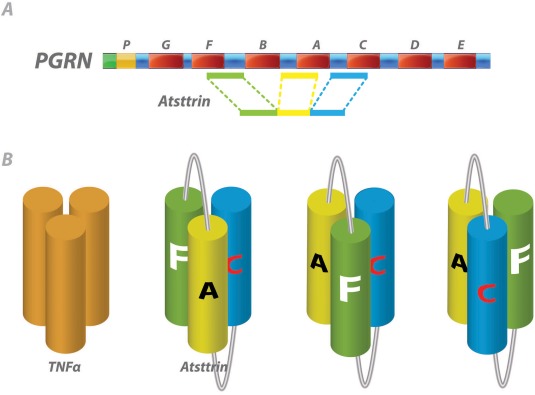
**A,** Domain structure and organization of progranulin (PGRN) and Atsttrin. PGRN consists of 7.5 repeats of a cysteine‐rich granulin motif in the order of P‐G‐F‐B‐A‐C‐D‐E, where A–G are full repeats and P is the half motif. Atsttrin, derived from PGRN, consists of 3 half units of granulins A, C, and F and their accompanying linker regions. **B,** Proposed models for explaining the independent action of 3 tumor necrosis factor receptor (TNFR)–binding domains of PGRN. TNF trimers bind to receptors in a heterohexameric 3:3 complex 88. The 3 fragments of Atsttrin interact independently with TNFR, and changing the order of these fragments does not affect the ability to bind to TNFR 15. It is proposed that each TNFR‐binding domain may function as a single TNF molecule, and the intact Atsttrin might resemble a TNF trimer through internal folding at the linker regions.

PGRN, also known as granulin‐epithelin precursor, proepithelin, acrogranin, and GP88/PC cell–derived growth factor, is a 593–amino acid autocrine growth factor. PGRN contains 7.5 repeats of a cysteine‐rich motif (CX5–6CX5CCX8CCX6CCXDX2HCCPX4CX5–6C) and forms a unique “beads‐on‐a‐string” structure 13. PGRN was first found to bind to TNFR in a yeast‐2‐hybrid screening for PGRN‐binding proteins 14. The interaction was subsequently validated in human cells. Surface plasmon resonance analysis revealed that PGRN bound to both TNFRI and TNFRII and with greater affinity than TNF to TNFRII 8, 14. Three fragments of PGRN and their adjacent linkers enable the ligand to bind to TNF receptors 15. Notably, PGRN showed therapeutic effects in several models of TNF‐mediated inflammatory arthritis, including collagen‐induced arthritis (CIA), collagen antibody–induced arthritis, and spontaneous arthritis in the TNF‐transgenic mouse model 14, 16, 17. Furthermore, a novel PGRN mimetic called Atsttrin (Figure 1) had a more pronounced beneficial effect than PGRN in inflammatory arthritis 14. Currently marketed anti‐TNF therapies bind to the TNF ligand; in contrast, Atsttrin binds to TNFR and not to TNF itself. Atsttrin was more efficacious than current anti‐TNF therapies, including etanercept, in several preclinical inflammatory arthritis models tested 14.

Accumulating evidence indicates that TNF orchestrates OA pathology 18. Recent findings support the notion that PGRN could also modulate the etiopathogenesis of OA. PGRN is an important regulator of cartilage development 19, 20 and was identified as an OA‐associated growth factor in a genome‐wide screen for differentially expressed genes in OA 21, and its deficiency in aging mice led to a spontaneous OA‐like phenotype characterized by severe breakdown of cartilage structure 22. The OA‐like pathology was attenuated by the local delivery of a recombinant PGRN protein. Intraarticular transplantation of Atsttrin‐transduced mesenchymal stem cells inhibited TNF‐mediated catabolic response, ameliorating OA development 23. One chondroprotective mechanism has been proposed, namely, that PGRN increases the levels of anabolic biomarkers and suppresses the inflammatory action of TNF in cartilage and chondrocytes via activation of the ERK‐1/2 signaling pathway 19.

The direct impact of PGRN on bone remodeling has yet to be determined, with current knowledge derived from a model of bone healing. In mice at least, PGRN deficiency delayed bone healing, while recombinant PGRN enhanced bone regeneration 24. Furthermore, PGRN‐mediated bone formation was dependent upon TNFRII but not TNFRI. In that same study, Zhao et al showed that PGRN blocked osteoclastogenesis in TNF‐transgenic mice. Taken together, these findings imply that PGRN exerts dual action on bone during inflammatory arthritis, first, by inhibiting TNF‐induced bone erosion by osteoclasts, and second, by promoting osteoblast‐dependent mineral apposition via TNFRII. Findings of a recent study using Atsttrin incorporated into 3‐dimensional–printed alginate/hydroxyapatite scaffolds imply that PGRN stimulates bone regeneration by inhibiting TNF signaling 25.

The inflammatory and catabolic actions of TNF are largely mediated through its interaction with TNFRI. However, we continue to have limited understanding of the impact of TNFRII‐mediated signaling. Recent studies indicate that TNFRII signaling is beneficial and protects against joint destruction 26, 27. Studies also reveal differential roles of TNFRI and TNFRII in PGRN‐mediated fracture healing and OA 22, 24, 28. Although PGRN and TNF exhibit comparable binding affinity to TNFRI, the binding affinity of PGRN for TNFRII is ∼600‐fold higher than that of TNF 14. Since PGRN and TNF compete for binding to the same extracellular cysteine‐rich domains (CRDs) of TNFR, CRD2 and CRD3 (8), PGRN acts as a naturally occurring antagonist of TNF and disturbs the binding of TNF to TNF receptors. More importantly, PGRN also acts as a ligand of TNFRII and directly activates the PGRN/TNFRII protective and antiinflammatory pathway. TNFRII has been shown to be critical for PGRN‐mediated protection in OA and bone fracture healing 22, 24, 28. A recent study showing that local injection of soluble TNFRII (sTNFRII; etanercept) resulted in more severe joint destruction in a mouse model of OA 29 also suggested the importance of PGRN‐mediated protection in OA. Injection of sTNFRII inhibits both TNF and PGRN. Furthermore, PGRN may be more inhibited than TNF, as the binding affinity of PGRN to TNFRII is much higher than that of TNF.

Unlike etanercept, mouse monoclonal antibody to TNF (infliximab) and humanized monoclonal antibody to TNF (adalimumab) are specific for TNF and have been shown to be protective against OA in animal models 30. The opposite effects of TNF‐specific (i.e., infliximab and adalimumab) and nonspecific (i.e., etanercept) inhibitors in OA indicate the critical protective role of other ligand(s) of TNFR (i.e., PGRN) in the pathogenesis of OA 31. Thus, future studies are warranted to clarify the complex interplay between TNF, PGRN, and their receptors in the pathogenesis of arthritis and bone remodeling, which not only will improve our understanding of TNFR signaling in the pathogenesis of these musculoskeletal disorders but also may lead to innovative therapies via selective targeting of distinct TNFR pathways.

## TL1A–DR‐3 interactions in arthritis and bone remodeling

DR‐3 (TNFRSF25, Apo‐3, lymphocyte‐associated receptor of death, TNFR‐like molecule 3, TNFR‐related apoptosis‐mediating protein, WSL‐1) was discovered simultaneously by multiple groups in the middle‐to‐late 1990s, when a combination of BLAST homology searches to Fas and TNFRI 32, 33 and a yeast‐2‐hybrid library screening using a TNFRI death domain as bait 34 identified a closely related protein. Subsequently, DR‐3 emerged as the closest structural homolog to TNFRI, containing an equivalent 4 CRDs as well as an intracellular death domain. However, unlike TNFRI, whose cellular distribution is widespread and whose surface expression can be controlled by the generation of soluble forms through cleavage, DR‐3 has a more restricted tissue distribution and is regulated by the expression of multiple activation‐induced splice variants, including soluble and death domain–containing transmembrane forms with excision of the membrane‐proximal CRD 33, 35. The exact function of these splice variants remains unclear.

The identification of ligand(s) for DR‐3 has been complicated by the number of potential candidates and their altering nomenclature 36, but prior to the discovery of PGRN, one TNFSF member, TL1A (TNFSF15) 37, had withstood stringent biochemical and functional scrutiny for DR‐3 specificity 10, 38. TL1A is the product of a longer alternative messenger RNA transcript for a protein initially named vascular endothelial growth inhibitor (TL1), so named for its capacity to inhibit angiogenesis and induce apoptosis of endothelial cells 39. As its name and nomenclature suggest, TL1A is closely related in structure to TNF, encoding a type II transmembrane protein with a metalloproteinase cleavage site allowing release of a soluble molecule, but it also has distinct expression patterns as it is found in ng/ml concentrations in serum from healthy individuals 40, which suggests that it has physiologically different levels of production and functional regulation. In this regard, there may also be significant differences between species, as decoy receptor 3, the decoy ligand for LIGHT (TNF ligand superfamily member 14), TL1A, and FasL, is found only in humans and not in mice. It is in this context that the function of DR‐3 and its potential for therapy should be interpreted.

The generation of transgenic mice genetically deficient for DR‐3 or TL1A or overexpressing TL1A or dominant‐negative forms of DR‐3 has given rise to many in vivo studies describing the essential requirement for the DR‐3/TL1A pathway in models of multiple autoimmune and inflammatory diseases. These have supported an ever‐growing list of in vitro human functional and genetic studies that have associated DR‐3 and TL1A with human diseases ranging from inflammatory bowel disease (IBD) and primary biliary cirrhosis to leprosy (comprehensively reviewed in ref. 41). Of significance for this review were findings that suggested alternate respective ligands for DR‐3 and TL1A. This included the apparent greater protection against experimental autoimmune encephalomyelitis afforded to DR‐3^−/−^ mice 38 compared to TL1A^−/−^ mice 42 in otherwise similar models of disease and the DR‐3–independent triggering of TNFRII expression by TL1A in kidney organ cultures 43. The underlying conclusion was that there were still unknown interactions for this complex of proteins, which would have to be discovered and dissected in detail before their full potential as therapeutic targets could be understood.

With specific regard to disorders of bone, initial genetic studies suggested that DR‐3 gene duplication 44 and a mutation predicted to destabilize DR‐3 (45) were linked to development of RA, while synovial cells from RA patients exhibited a hypermethylated DR‐3 gene suggestive of activation 46; however, genome‐wide association studies have had less success with supporting this connection. Two early investigations associated genetic variation around the DR‐3 (TNFRSF25) locus with RA 47, 48, but more recent ones have not. In contrast, genetic variation at the TL1A (TNFSF15) locus has not been associated with RA but has been linked to another bone disorder, ankylosing spondylitis 49. Regardless, increased levels of TL1A have been reported in the serum of patients with both of these arthritides 40, 50, 51 as well as in the synovial tissue and synovial exudates of rheumatoid factor–positive RA patients 52, 53.

The functional consequences of raised TL1A levels in these disorders have generally been associated with a range of outcomes that depend on the type and differentiation state of the DR‐3–expressing cell to which TL1A is binding and signaling. In this review, we will cover those cell types specifically associated with bone physiology irrespective of the context of inflammation, although it should be noted that there may also be secondary effects as TL1A can induce TNF 54, thereby having the capacity to trigger a broad range of secondary effects associated with other proinflammatory cytokines. The DR‐3/TL1A axis was first described as a T cell costimulator 37, but its effects on Th17 cells, which are drivers of osteoclastogenesis and therefore inflammatory bone resorption 55, highlighted the complexity in the outcome of TL1A signaling. Initial reports in TL1A^−/−^ mice suggested that TL1A regulated Th17 cell differentiation 42, but more extensive in vitro studies in both DR‐3^−/−^ mice 56 and healthy human subjects indicated that Th17 cell differentiation from naive CD4+ T cells was impaired by TL1A, while maintenance of the response once T cells were committed to becoming Th17 cells was enhanced by TL1A 57. Intriguingly, recent studies have shown that TL1A‐driven Th17 cell differentiation from naive CD4+ T cells occurs in samples from RA patients 51, 58. Why these differences have been observed remains an area of debate, although the underlying theme is that TL1A promotes the Th17 cell response in RA.

The development of the main effectors of bone resorption, osteoclasts, is also regulated by the DR‐3/TL1A axis, at least in a setting of inflammation. While osteoclastogenesis driven by macrophage colony‐stimulating factor and RANKL was unaffected in DR‐3^−/−^ mice, these animals exhibited resistance to cartilage destruction and bone erosion in a model of antigen‐induced arthritis (AIA) 10, 59. Furthermore, DR‐3^−/−^ mice were resistant to exacerbation of disease induced by exogenous addition of TL1A to DR‐3^+/+^ animals, while antagonism of the pathway with anti‐TL1A monoclonal antibody ameliorated disease in CIA 10. Addition of exogenous TL1A also exacerbated CIA 53. The direct nature of this signaling in myeloid cells has been demonstrated, with DR‐3 expression being induced during the process of macrophage differentiation and TL1A signaling resulting in DR‐3–dependent production of the gelatinase matrix metalloproteinase 9 60. The DR‐3/TL1A pathway may also control other aspects of macrophage differentiation that promote the arthritis process. Thus, DR‐3 regulates the expression of scavenger receptors on macrophages 61, which have been implicated in AIA‐induced cartilage destruction 62.

Finally, DR‐3 also modulates osteoblast function. Human osteoblast cell lines were first reported to express DR‐3 in 2003 63; these cell lines were then used to demonstrate differential regulation dependent on cell culture conditions. Crosslinking induced apoptosis at low density but differentiation at high density 11. The subsequent reported association between TL1A and ankylosing spondylitis 49 and the breeding of the DR‐3^−/−^ mouse genotype on a DBA/1 background, which results in a mouse that spontaneously develops ankylosing enthesopathy 64, led to a recent study of the role of DR‐3 in osteoblast function in vitro and in vivo. Indeed, DBA/1 DR‐3^−/−^ mice showed significantly less thoracic spine–specific bone formation in vivo, while DR‐3^−/−^ mouse osteoblast cultures exhibited reduced levels of alkaline phosphatase, osteopontin, and mineral apposition 12. Thus, the DR‐3/TL1A axis is involved in the direct regulation of every major cell type involved in bone physiology, and recent data 64 suggest that it has an important homeostatic role in this tissue in addition to its more established function in inflammatory disease.

## PGRN–DR‐3 interactions in arthritis and bone remodeling

Screening the associations of Atsttrin with all members of the TNFR subfamily led to the discovery that in addition to TNFR, PGRN/Atsttrin directly binds to DR‐3 and inhibits TL1A activity 65. Structural modeling of DR‐3 predicts a structure similar to that of TNFRI in which primary contacts with TL1A are in the second and third CRD 45. In addition, a mutation linked to RA at the end of CRD3 is in a region critical for structural integrity of ligand–receptor complexes 45. The first 3 CRD domains of the extracellular portion of DR‐3 (i.e., CRD1, CRD2, and CRD3) are all required for interacting with Atsttrin. PGRN was also found to directly bind to DR‐3 in an in vitro binding assay, as it did to TNF receptors 65. Atsttrin inhibited TL1A‐stimulated expression of TL1A target genes C1qTNF3 and βigH3 in a dose‐dependent manner. In addition, Atsttrin effectively neutralized TL1A‐promoted osteoclastogenesis in vitro 65.

The association of PGRN with TNFR and DR‐3 also led to investigations of the immunologic mechanisms underlying PGRN‐mediated antiinflammatory and protective activities in autoimmune diseases 66, 67, 68. Since both animal and human studies have demonstrated that Treg cells play a critical role in the prevention of autoimmunity and other pathologic immune responses, the effects of PGRN on Treg cell differentiation and function were first determined.

PGRN protects Treg cells from a negative regulation by TNF, and these protective effects are primarily mediated by TNFRII 67, 68. In contrast, antibodies to recombinant PGRN led to an increase in TNF‐induced down‐regulation of FoxP3 in CD4+CD25^high^ Treg cells 69. In addition, PGRN was able to stimulate the conversion of CD4+CD25− T cells into induced Treg cells in a dose‐dependent manner in vitro. Further, PGRN showed synergistic effects with transforming growth factor β1 on the induction of Treg cells 68. PGRN was required for the immunosuppressive function of Treg cells, since PGRN‐deficient Treg cells have significantly decreased ability to suppress the proliferation of effector T cells. PGRN deficiency caused a marked reduction in Treg cell numbers in the course of inflammatory arthritis 68. In a bone marrow chimera and CD4+CD45RB^high^ T cell transfer model, lack of PGRN signaling in CD4+ T cells also exacerbated experimental colitis. In addition, PGRN‐mediated protective effect was compromised in the absence of interleukin‐10 (IL‐10) or TNFRII signaling 67. It is noted that PGRN‐mediated regulation of Treg cells appears to be inflammation dependent, because PGRN deficiency does not alter the numbers of CD4+CD25+FoxP3+ Treg cells in vivo under physiologic conditions 68. PGRN inhibits expression and release of the chemokines CXCL9 and CXCL10 in a TNFRI‐dependent manner in CD4+ T cells 66.

The DR‐3 pathway may also contribute to PGRN‐mediated protective effect in inflammatory diseases, since a recent study showed that agonistic antibody to DR‐3 expanded CD4+FoxP3+ Treg cells in vivo, which in turn suppressed immune responses 70. In addition, a neuropathology develops with age in both DR‐3^−/−^ 71 and PGRN‐deficient 72 mice. Intriguingly, transgenic overexpression of TL1A in both the myeloid and T cell lineage results in in vivo expansion of Treg cells, although these eventually become dysregulated and intestinal inflammation develops 10.

**Figure 2:**
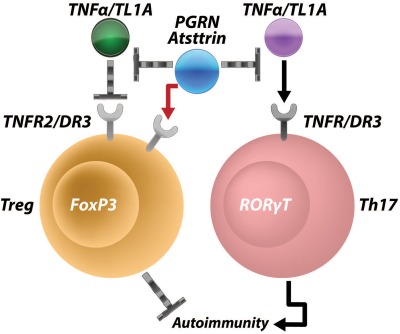
Proposed model illustrating the multiple signaling pathways by which progranulin (PGRN) and its derivative Atsttrin exert their protective actions in autoimmunity. PGRN (or Atsttrin) binds to tumor necrosis factor receptor type II (TNFRII) and stimulates the formation and function of Treg cells, but may antagonize TNF‐like molecule 1A (TL1A)/death receptor 3 (DR‐3) signaling in these cells. PGRN (or Atsttrin) also antagonizes TNF/TNFRI and TL1A/DR‐3 signaling and inhibits their inflammatory activities. RORγt = retinoic acid receptor–related orphan nuclear receptor γt.

In contrast to Treg cells, the frequency of Th17 cells was significantly decreased in spleens of mice treated with recombinant PGRN in a CIA model 67, 68. In addition, the serum IL‐17 level was also significantly decreased in PGRN‐treated mice. Further, both TNFRI and DR‐3 pathways were found to be involved in PGRN inhibition of IL‐17–producing cells. Taken together, PGRN and its Atsttrin mimetic appear to exert their antiinflammatory activities through multiple pathways: 1) by activation of the PGRN/TNFRII protective pathway and 2) by inhibition of TNF/TNFRI and TL1A/DR‐3 inflammatory signaling (Figure 2).

## Clinical perspective

Because TNF is one of the key main mediators of inflammation, it is no surprise that alterations of its physiologic antagonist PGRN have a direct impact on the initiation and progression of arthritis. The effect of TNF antagonism by PGRN should be at least comparable to that of conventional TNF blockers 14. The additional specific inhibition of the TL1A–DR‐3 interaction and the activation of the TNFRII antiinflammatory pathway by PGRN or its derivate 65 are unique characteristics and might represent a significant advantage over conventional TNF inhibitors, particularly for patients with refractory or relapsing disease who are taking conventional TNF blockers. Blocking the TL1A–DR‐3 interaction probably offers additional positive effects through reduction of proinflammatory cytokines, reduction of autoantibody formation, and reduction of osteoclastogenesis 10, 53.

A potential disadvantage of PGRN or Atsttrin compared to anti‐TNF antibodies might be that anti‐TNF antibodies can trigger apoptosis of proinflammatory T lymphocytes by binding to membranous TNF. This effect, which is also missing for TNFR‐Fc fusion proteins, appears to play a particular role in inflammatory bowel diseases and less of a role in arthritis 73. The question is whether administration of PGRN or a derivative confers a higher risk of iatrogenic induced neoplasms than administration of conventional TNF blockers. Use of conventional TNF blockers results in an elevated risk of reactivating latent infections such as *Mycobacterium tuberculosis* or viral hepatitis, or of developing opportunistic infections 74. The effects of administered recombinant PGRN or its derivative on the risk of opportunistic infections remain a subject of speculation and are not discussed further in this review.

Another question arises from the discovery of autoantibodies to PGRN. Can recombinant PGRN or Atsttrin be administered to patients with preexisting antibodies to PGRN? Frequently occurring anti‐PGRN antibodies have been identified in a wide spectrum of autoimmune diseases including RA and, surprisingly, psoriatic arthritis, which had been regarded as a seronegative disease 5, 75. Antibodies to PGRN occur in relevant titers, belong predominantly to the IgG1 subclass (also IgA in IBD), and have a neutralizing effect on plasma PGRN levels, and thus are likely to act in a proinflammatory manner.

Epitope mapping identified a binding region within the N‐terminal 112 amino acids of PGRN as a target of antibodies to PGRN in all patients. This means that autoantibodies to PGRN target the antiinflammatory PGRN and possibly cotarget only mature granulin G, the most N‐terminal granulin motif. Despite the structural similarity of granulin G and the other 6 granulins, no binding was detected against granulin motifs other than granulin G 75. With regard to Atsttrin, no antibodies have been detected so far that are directed against those parts of PGRN that are constitutive of Atsttrin (i.e., granulin F, granulin A, granulin C, and the appropriate linker regions) 14. Nevertheless, epitope spreading and immunogenicity should be monitored closely in preclinical and clinical trials addressing the therapeutic effects of Atsttrin administration. To our knowledge, a potential binding of patient‐derived, preexisting antibodies to PGRN against Atsttrin itself has not yet been tested, and this possibility should be tested for and excluded.

**Figure 3:**
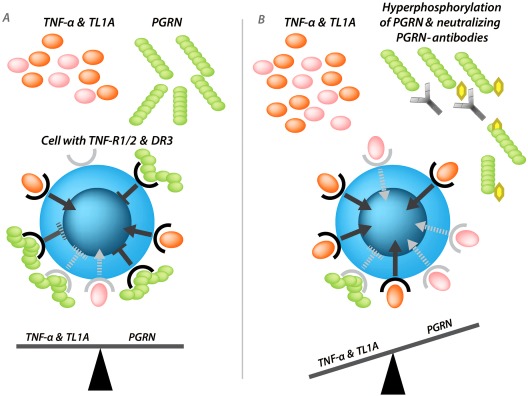
**A,** Balance of tumor necrosis factor (TNF) and TNF‐like molecule 1A (TL1A) and their antagonist progranulin (PGRN) in a healthy control. **B,** Dysbalance of proinflammatory TNF and TL1A and antiinflammatory PGRN due to overexpression of proinflammatory TNF and TL1A and diminished antagonistic effects of PGRN due to hyperphosphorylation of PGRN at Ser^81^ and induction of neutralizing antibodies to PGRN. TNF‐R1/2 = TNF receptors type I and type II; DR‐3 = death receptor 3.

As a reason for the breakdown of self‐tolerance against PGRN, a second immunogenic PGRN isoform, hyperphosphorylated at Ser^81^, was identified exclusively in an anti‐PGRN antibody–positive patient 76. This hyperphosphorylated PGRN is caused by inactivated protein phosphatase 1. Interestingly, phosphorylation of PGRN at Ser^81^ prevents interaction with TNFRI, TNFRII, and DR‐3, so hyperphosphorylated PGRN has lost its antiinflammatory function. Considering these facts, it seems that a reasonable therapeutic strategy would be to compensate for the imbalance of pro‐ and antiinflammatory molecules due to lack of functional PGRN (caused by anti‐PGRN antibodies or hyperphosphorylation of PGRN at Ser^81^) and/or excessive secretion of TNF and TL1A by administering a recombinant PGRN derivate that cannot be neutralized by preexisting autoantibodies to PGRN (Figure 3).

In conclusion, PGRN and its interaction with TNF/TNFRI/TNFRII and TL1A/DR‐3 represent attractive new therapeutic targets (Table 2). When we consider the underlying theory and the known preclinical data, Atsttrin could be a therapeutic alternative in cases of refractory or recurrent arthritis.

**Table 2:** Summary of key points about PGRN and TNFR and DR‐3 pathways in RA, OA, SpA, and other arthropathies^a^

Key points	References
PGRN Also known as granulin‐epithelin precursor, proepithelin, acrogranin, and GP88/PC cell–derived growth factor Autocrine growth factor with 593 amino acids Contains 7.5 repeats of a cysteine‐rich motif Involved in embryogenesis, wound healing, countering inflammation, host defense, acting as neurotrophic factor High levels associated with several human cancers	14, 16, 17
PGRN as ligand of TNFRI, TNFRII, and DR‐3 PGRN acts as ligand of TNFRI, TNFRII, and DR‐3 and as physiologic antagonist of TNF, LTα, and TL1A Inhibits TNFRI and DR‐3 pathways, but activates TNFRII pathway Binding affinity of PGRN for TNFRII is ∼600‐fold higher than that of TNF PGRN affinity for TNFRI, TNFRII, and DR‐3 originates from granulins F, A, and C with linker regions Atsttrin is smallest recombinant derivate of PGRN and is synthesized from granulins F, A, and C and linker regions P3, P4, and P5 of PGRN with preserved antiinflammatory effect PGRN attenuates TNF‐induced down‐modulation of CD4+CD25^high^FoxP3+ Treg cells PGRN stimulates conversion of CD4+CD25− T cells into induced Treg cells	14, 65, 68
PGRN, TNFRI, and TNFRII in OA Low PGRN levels yield spontaneous OA High PGRN levels yield anabolic function Catabolic effect of TNF is mainly mediated via TNFRI TNFRII pathway is both antiinflammatory and osteoprotective Administration of sTNFRII‐Fc fusion protein neutralizes TNF and PGRN and leads to exacerbation of OA Administration of anti‐TNF monoclonal antibodies neutralizes TNF specifically and ameliorates OA PGRN accounts for the opposite effects of sTNFRII‐Fc fusion protein and anti‐TNF monoclonal antibodies	30, 31
TL1A/DR‐3	10, 51, 58, 59
High levels of TL1A induce Th17 cell response in RA DR‐3^−/−^ mice are resistant to cartilage destruction in AIA CIA is exacerbated by TL1A and ameliorated by anti‐TL1A monoclonal antibody TL1A/DR‐3 activation induces MMP‐9 and CCL3 Decoy receptor 3 decoy ligand for TL1A, FasL, and LIGHT is found only in humans and not in mice, making results from mouse models difficult to translate	
PGRN isoform hyperphosphorylated at Ser^81^ and anti‐PGRN antibodies Neutralizing antibodies directed against a binding region within the N‐terminal 112 amino acids of PGRN occur frequently in various autoimmune diseases Anti‐PGRN antibodies are induced by a second, transiently occurring PGRN isoform hyperphosphorylated at Ser^81^ PGRN isoform hyperphosphorylated at Ser^81^ lacks affinity for TNFRI, TNFRII, and DR‐3 and thus antagonizes TNF and TL1A These phenomena result in dysbalance of proinflammatory TNF and TL1A and antiinflammatory functional PGRN in various inflammatory diseases	5, 74, 75
Clinical perspective Targeting of TNFRSF and TNFSF is a common therapeutic strategy There are possible advantages of using PGRN or Atsttrin instead of conventional TNF blockers due to additional inhibition of DR‐3 and activation of TNFRII Autoantibodies to PGRN regularly target the binding region within the N‐terminal 112 amino acids and not the parts of PGRN that are constitutive of Atsttrin; however, their affinity for Atsttrin has not been excluded Risk of side effects concerning susceptibility to infectious diseases, emergence of new autoimmune phenomena, or cancer remains unclear	79

a PGRN = progranulin; TNFR = tumor necrosis factor receptor; DR‐3 = death receptor 3; RA = rheumatoid arthritis; OA = osteoarthritis; SpA = spondyloarthritis; LTα = lymphotoxin α; TL1A = TNF‐like molecule 1A; sTNFRII = soluble TNFRII; AIA = antigen‐induced arthritis; CIA = collagen‐induced arthritis; MMP‐9 = matrix metalloproteinase 9; LIGHT = TNF ligand superfamily member 14; TNFRSF = TNFR superfamily.

## AUTHOR CONTRIBUTIONS

All authors were involved in drafting the article or revising it critically for important intellectual content, and all authors approved the final version to be published.
